# Genome-wide analysis of rice *cis*-natural antisense transcription under cadmium exposure using strand-specific RNA-Seq

**DOI:** 10.1186/s12864-017-4108-5

**Published:** 2017-10-06

**Authors:** Youko Oono, Takayuki Yazawa, Hiroyuki Kanamori, Harumi Sasaki, Satomi Mori, Takashi Matsumoto

**Affiliations:** 1 Institute of Crop Science, National Agriculture and Food Research Organization, 2-1-2 Kannondai, Tsukuba, Ibaraki, 305-8602 Japan; 2 Present address: Laboratory of Plant Molecular Breeding, Department of Bioscience, Tokyo University of Agriculture, 1-1-1 Sakuragaoka, Setagaya-ku, Tokyo, 156-8602 Japan

**Keywords:** Strand-specific RNA-Seq (ssRNA-Seq), *cis*-natural antisense transcript (*cis*-NATs), Cadmium (Cd), Abiotic stress, Rice, Transcriptome

## Abstract

**Background:**

The elucidation of novel transcripts and their expression in response to various stress conditions is necessary to understand the transcriptional network of plants as an adaptation to biotic and abiotic stresses. We performed strand-specific RNA-Seq (ssRNA-Seq) on rice exposed to cadmium (Cd) for 24 h and investigated the expression of *cis*-natural antisense transcripts (*cis*-NATs), a class of endogenous coding or non-protein-coding RNAs with sequence complementarity to the opposite strands of RAP transcripts.

**Results:**

Many RAP transcripts possessed *cis*-NATs and these *cis*-NATs were responsive to some extent. *Cis*-NATs were upregulated from 26, 266 and 409 RAP gene loci, while 2054, 2501 and 2825 RAP transcripts were upregulated from 38,123 RAP loci under high Cd exposure in roots at 1, 12 and 24 h, respectively. In addition, most of the upregulated *cis*-NATs showed little upregulation under ABA or cold treatment. A number of *cis*-NATs were upregulated from less than 35 RAP gene loci in different tissue and time-point combinations under low Cd exposure, suggesting that *cis*-NATs respond to environmental stress. Furthermore, 409 RAP transcripts with upregulated *cis*-NATs were classified into three groups based on the expression of the RAP transcripts from the opposite DNA strand, including 138 upregulated, 128 invariable, and 143 downregulated transcripts, although the responses of *cis*-NATs and RAP transcripts were not always correlated.

**Conclusions:**

We have shown that the *cis*-NATs identified by ssRNA-Seq analysis are novel genes and that some of them are stress-specific and show different responses depending on the degree of stress and tissue. These results improve our understanding of the complete molecular mechanism of plant adaptation to Cd exposure.

**Electronic supplementary material:**

The online version of this article (10.1186/s12864-017-4108-5) contains supplementary material, which is available to authorized users.

## Background

Rice (*Oryza sativa* L.) is a major staple in many parts of the world. The transcriptomic network of rice under various environmental stress conditions remains to be fully elucidated despite many studies based on RNA sequencing technology, which can accurately quantify gene expression levels over a broad dynamic range and detect transcripts expressed at very low or very high levels, as well as subtle changes [1–3]. This is because of the complexity of the transcriptome including the existence of *cis*-natural antisense transcripts (*cis*-NATs), a class of endogenous coding or non-protein-coding RNAs with sequence complementarity to the opposite strand of an annotated gene. In addition, many genes have not been functionally characterized, and transcription site boundaries and transcript structures can sometimes change; for example, because of splice isoforms and editing [4, 5]. The set of transcripts present in a cell, tissue, organ or whole organism also varies at different points in time, and may change depending on the developmental stage or environmental conditions.

We performed a time-course transcriptome analysis of rice (*Oryza sativa ssp. japonica* cv. Nipponbare) under 140 environmental stress and plant hormone treatment conditions using the RNA-Seq platform to elucidate the complete set of transcripts including transcripts showing subtle changes in expression, rare transcripts and variants. The transcripts showing diverse responses under each condition were compiled in the TENOR (Transcriptome ENcyclopedia Of Rice, http://tenor.dna.affrc.go.jp) database [6]. They included a large number of novel transcripts (unannotated transcribed regions with no overlap to any known locus) identified by comparing known transcript structures annotated in the RAP-DB (http://rapdb.dna.affrc.go.jp/) and the MSU Rice Genome Annotation Project database (http://rice.plantbiology.msu.edu/) with Cufflinks-predicted structures. The RNA-Seq technology therefore allows for ultra-deep and highly parallel sequencing of basal transcriptomes under stress conditions and can overcome several limitations of microarray technology.

Recently, strand-specific RNA-Seq (ssRNA-Seq) has become a useful method to define transcriptional orientations and to assess the presence of *cis*-natural antisense transcripts (*cis*-NATs). As RNA-Seq analysis does not recognize transcripts transcribed from the opposite DNA strand of the same genomic locus that overlap partly with sense RNA in the same or opposite orientation [7], *cis*-NATs may have not been identified at all. Large-scale analysis indicates thousands of *cis*-NATs respond to light in *Arabidopsis* [8] and heat in *Brassica rapa* [9], and are expressed in rice under salt, drought and cold stresses, and normal conditions [7]. A few *cis*-NATs have been reported to act as a regulatory class of RNA and can affect transcription or translation by various mechanisms including transcriptional interference. Upregulation of the antisense FLC (FLOWERING LOCUS C, floral repressor gene) transcript may be part of the cold-sensing mechanism, which is correlated with downregulation of the *FLC* transcript [10]. Downregulation of antisense *PHO1;2* (PHOSPHATE1;2) transcript expression resulted in a decrease in the PHO1;2 protein, while constitutive overexpression of the antisense *PHO1;2* transcript led to a strong increase of PHO1;2, even under phosphate-sufficient conditions, suggesting the *cis*-NAT acts as a translational enhancer for the sense mRNA in rice phosphate homeostasis [11]. However, the existence and responses of *cis*-NATs as novel transcripts are still not fully understood under various stress conditions.

In the present study, we took advantage of the ssRNA-Seq method to deeply sequence cDNAs from transcribed RNAs with a clear transcriptional orientation in rice that were associated with the specific stress response to Cd exposure. To our knowledge, this is the first report of the existence of genes encoding *cis*-NATs responsive to Cd stress. Only loci overlapping with sense DNA annotated RAP (Rice Annotation Project: http://rapdb.dna.affrc.go.jp/) genes were used to search for *cis*-NATs. We focused on *cis*-NATs from RAP gene loci and excluded the loci of sense-antisense RAP genes, which are transcribed bidirectionally from an overlapping genomic region. Our aim was to explore the existence of *cis*-NATs, which are generally rare, via ssRNA-Seq and to identify novel transcripts expressed specifically under Cd exposure.

## Methods

### Plant culture and treatments

Rice (*Oryza sativa ssp. japonica* cv. Nipponbare) seeds were germinated and grown by hydroponic culture in medium containing 1.425 mM NH_4_NO_3_, 0.323 mM NaH_2_PO_4_, 0.513 mM K_2_SO_4_, 0.998 mM CaCl_2_, 1.643 mM MgSO_4_, 0.009 mM MnCl_2_, 0.075 mM (NH_4_)_6_ Mo_7_O_24_, 0.019 mM H_3_BO_3_, 0.155 mM CuSO_4_, 0.036 mM FeCl_3_, 0.070 mM citric acid, and 0.152 mM ZnSO_4_ [12]. The seedlings were grown in a growth chamber at 28 °C under a 16 h light/8 h dark cycle with the light period from 6:00 AM to 10:00 PM for 10 days. For Cd exposure, the seedlings were transferred to a similar culture medium with 0.2, 1 or 50 μM CdSO_4_. For ABA treatment, the seedlings were transferred to culture medium with 100 μM ABA. The plants were maintained under each condition for 24 h at 28 °C with a 16 h light/8 h dark cycle. For cold treatment, the seedlings were transferred to a growth chamber set at 4 °C. Root and shoot samples were collected, frozen in liquid nitrogen, and stored at −80 °C until subsequent analyses.

### Sequencing and mapping of short reads onto the rice genome

Total RNA was extracted from all samples using a QIAGEN kit (Invitrogen, Carlsbad, CA, USA) according to the manufacturer’s instructions. Strand-specific RNA-Seq libraries were prepared following the “Directional mRNA-Seq Library Prep Pre-Release” protocol by Illumina. Each sample was sequenced for 75 cycles on an Illumina GAIIx. Low-quality bases (<Q15) were trimmed from both the 5′- and 3′-ends of the reads until a stretch of three or more high-quality (≥Q15) nucleotides appeared using an in-house program. Trimming of Illumina adaptor sequences was performed by Cutadapt [13] version 0.9.4. To remove reads from rRNAs, we aligned the reads against rice rRNA gene sequences downloaded from the Rice Annotation Project DataBase (RAP-DB) [14] using Bowtie [15] version 0.12.7, and removed any matching reads. The resulting reads were aligned to the Os-Nipponbare-Reference-The International Rice Genome Sequencing Project-1.0 genome assembly (IRGSP-1.0) (http://rapdb.dna.affrc.go.jp/) using Bowtie, SAMtools version 0.1.18 [16], and TopHat [17] version 1.4.0. The expression level for each transcript was calculated as RPKM (Reads Per Kilobase exon Model per Million mapped reads) [18] based on the number of uniquely mapped reads using an in-house program.

### Analysis of gene ontology (GO) terms and responsive transcripts under stress treatment

The GO terms assigned to each transcript were obtained from the RAP-DB for each GO category under the biological process, molecular function, and cellular component domains. Differentially expressed transcripts were detected using a G-test with a FDR (False Discovery Rate) threshold of 0.1%. We focused on genes that had no overlapping genes in the opposite orientation. RPKM values were calculated from at least two biological replicate samples under Cd exposure with >0.9 correlation between them.

## Results and discussion

### ssRNA-Seq provides details of the transcriptional structures of *cis*-NATs transcribed bidirectionally from RAP loci

**Fig. 1:**
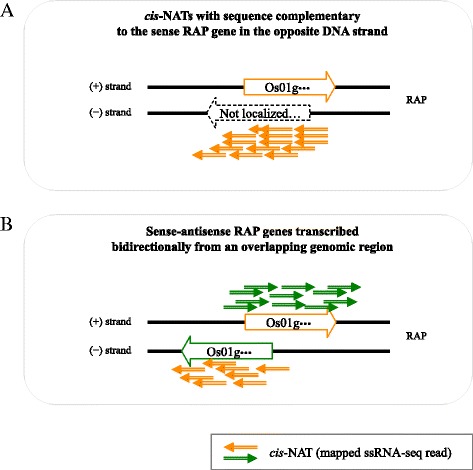
*cis*-NATs with sequence complementary to a RAP gene on the opposite DNA strand (**a**) and sense-antisense RAP genes transcribed bidirectionally from an overlapping genomic region (**b**). The mapped ssRNA-Seq reads of *cis*-NATs with sequence complementary to a RAP gene on the opposite DNA strand are indicated by arrows. The origins of reads from overlapping sense-antisense RAP gene loci were difficult to define

We generated transcriptome profiles of the early response to 0.2, 1 and 50 μM Cd exposure during plant growth to identify *cis*-NATs as novel transcripts using ssRNA-Seq, specifically at 1, 12 and 24 h. We also generated transcriptome profiles under control conditions at 0, 1, 12 and 24 h. In this study, we focused on *cis*-NATs with sequence complementary to a RAP gene on the opposite DNA strand to identify *cis*-NATs clearly (Fig. 1a), excluding sense-antisense genes that were transcribed bidirectionally from an overlapping genomic region (Fig. 1b). Because it is hard to distinguish antisense transcripts from sense transcripts transcribed from the same genomic strand in the overlapping genomic region, it can be difficult to accurately quantify the expression level.

### Rice genes responsive to Cd exposure

**Fig. 2:**
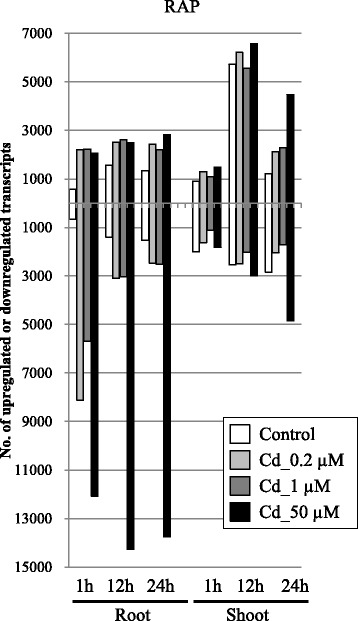
Distribution of upregulated and downregulated transcripts with RAP annotations under Cd exposure in roots (right) and shoots (left). RPKM fold changes at 1, 12 and 24 h were calculated for Cd-treated samples compared with non-treated samples (0 h). The total numbers of upregulated (upper) and downregulated (lower) transcripts in roots and shoots identified by ssRNA-Seq were determined by a G-test (FDR < 0.01) at each time point under 0.2 μM (light gray), 1 μM (dark gray), and 50 μM (black) Cd exposure, and in the control (white)

Cd exposure greatly affects rice growth and gene expression even at low concentrations [19]. For each set of conditions, an average of approximately 9.4 million (86.5%) quality-evaluated reads were mapped to the rice genome sequence and used for further analysis (Additional file 1: Table S1). Analysis of variance (G-test {FDR < 0.01}) on the RPKM-derived read counts was used to dissect the transcriptional responses associated with the time of collection (0, 1, 12 and 24 h), hydroponics type (control and Cd exposure) and tissue (root and shoot). In roots, the number of downregulated RAP transcripts was always greater than that of upregulated RAP transcripts (Fig. 2), which is the same tendency seen in previous results of RNA-Seq analysis [19]. In shoots, the number of downregulated transcripts gradually increased and reached a maximum under 50 μM Cd at 24 h. The number of responsive transcripts annotated by RAP at 24 h was 2864, 4906, 4734 and 16,567 in roots and 4043, 4155, 3990 and 9315 in shoots, after 0 (control), 0.2, 1 and 50 μM Cd exposure, respectively, compared with non-treatment (0 μM at 0 h) (Fig. 2), suggesting that 50 μM Cd exposure induced responses in more gene responses at 24 h. These data also suggest that the root was more affected by Cd stress than the shoot at 24 h, which is reasonable because roots are affected by Cd stress directly. Seventeen metal ion transporters were upregulated (>5-fold) under 50 μM Cd at 24 h (Table 1). The upregulation of seven genes was confirmed under Cd exposure by qRT-PCR analysis [19], suggesting ssRNA-Seq was successful in identifying Cd-responsive transcripts.

**Table 1:** Cadmium-upregulated metal ion transporters identified in rice

			Root							Shoot						
			Fold Change			RPKM				Fold Change			RPKM			
			Cd_50 μM			Control		Cd_50 μM		Cd_50 μM			Control		Cd_50 μM	
Family	Transcript	Length	1 h	12 h	24 h	0 h	1 h	12 h	24 h	1 h	12 h	24 h	0 h	1 h	12 h	24 h
HMA	*Os02t0585200-01*	797	94.7	280.1	**313.2**	5.0	568.4	1682.5	1881.4	2.5	4.3	4.6	3.8	11.3	19.9	21.0
	*Os03t0152000-01*	1400	132.8	239.5	**292.0**	8.0	1191.9	2149.8	2621.0	1.4	4.3	4.8	3.2	5.0	17.3	19.2
	*Os02t0585100-00*	646	15.9	54.7	**74.2**	19.8	329.9	1135.6	1539.8	1.6	4.6	**5.2**	0.9	2.0	7.5	8.7
	*Os02t0584800-01*	716	15.4	64.8	**70.1**	7.0	122.0	515.7	557.4	1.0	4.4	3.9	5.2	5.5	26.5	23.1
	*Os02t0584700-01*	642	8.8	20.1	**21.7**	14.3	134.4	306.4	331.1	1.1	2.1	2.6	8.5	9.6	19.2	23.7
	*Os04t0581800-01*	566	1.0	8.1	**17.5**	0.7	0.7	12.8	28.9	0.9	1.2	1.0	0.3	0.2	0.6	0.3
	*Os01t0976300-01*	821	4.3	11.3	**10.1**	40.8	178.5	471.7	421.3	0.5	2.4	4.5	7.0	3.1	17.9	35.2
	*Os03t0372600-00*	417	0.9	4.9	**9.9**	4.3	3.6	25.3	51.7	1.2	2.0	2.2	0.2	0.4	1.5	1.6
	*Os03t0111400-01*	546	0.5	1.1	1.5	58.8	30.7	63.3	88.8	0.7	3.1	**6.4**	12.9	9.1	41.8	87.6
MatE	*Os03t0188100-01*	1873	7.2	46.4	**36.5**	1.4	16.1	109.4	86.0	1.0	0.9	2.6	9.7	9.9	8.9	27.0
	*Os10t0344000-01*	1782	10.3	22.1	**14.9**	1.8	27.7	60.6	40.5	0.8	4.0	**15.5**	2.9	2.0	14.3	58.7
	*Os10t0345100-01*	1902	5.1	9.9	**7.5**	10.9	59.4	117.1	88.4	0.8	3.3	4.0	12.5	9.9	42.8	53.0
	*Os10t0344500-00*	1560	3.0	9.3	**7.1**	0.4	3.1	11.8	8.8	0.8	1.0	1.9	11.3	8.5	11.2	22.4
Zip	*Os03t0411800-01*	1332	0.4	0.7	2.0	7.3	2.1	4.9	16.0	1.5	3.5	**13.6**	9.8	15.0	36.6	146.0
PDR_assoc	*Os01t0609300-02*	1547	13.0	10.4	**7.1**	6.5	95.6	76.6	52.0	0.7	5.5	**9.5**	23.6	15.7	135.7	233.7
	*Os01t0609900-01*	4826	2.3	1.0	1.1	68.1	157.0	71.1	74.2	1.0	3.1	**5.3**	25.8	24.9	81.2	140.8
	*Os01t0609900-02*	4445	2.2	0.9	1.0	73.7	166.8	68.4	71.5	1.0	3.1	**5.3**	27.9	26.9	88.6	152.0

Bold characters show fold changes greater than 5 in upregulated transcripts

### Identification of *cis*-NATs with sequence complementary to a RAP gene on the opposite DNA strand

**Fig. 3:**
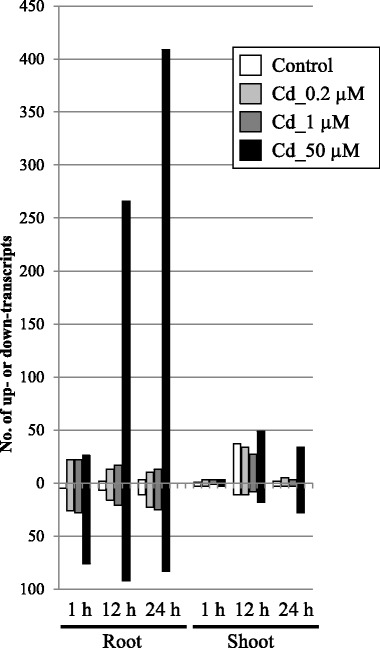
Distribution of RAP transcripts in roots and shoots with upregulated or downregulated *cis*-NATs under Cd exposure. RPKM fold changes at 1, 12 and 24 h were calculated for Cd-treated samples compared with non-treated samples (0 h). The total numbers of upregulated (upper) and downregulated (lower) transcripts in roots and shoots identified by RNA-Seq were determined by a G-test (FDR < 0.01) at each time point under 0.2 μM (light gray), 1 μM (dark gray), and 50 μM (black) Cd exposure, and in the control (white)

We focused on genomic loci containing a single RAP gene, which included 38,123 RAP gene loci, to define *cis*-NATs clearly among the 52,640 RAP genes (Fig. 1a). We investigated mapped reads with the opposite orientation in the overlapping genomic region. Among the genomic loci of the 38,123 RAP genes, reads were mapped to 29,323 RAP transcripts (88.0%) and *cis*-NATs that were transcribed bidirectionally from 15,588 RAP-overlapping transcripts (40.9%) in roots, and to 28,626 RAP transcripts (75.1%) and *cis*-NATs from 9851 overlapping transcripts (25.8%) in shoots at one time point at least under 0 (control), 0.2, 1 and/or 50 μM Cd exposure. This suggested that many single loci with RAP annotations were transcribed bidirectionally from an overlapping genomic region under Cd exposure, but the *cis*-NATs themselves were not supported by RAP. Approx. 30% of all annotated genes were shown to have significant antisense RNA expression in *Arabidopsis* [20], suggesting that *cis*-NATs are expressed to some degree in plants and may have diverse transcriptional regulatory mechanisms for carrying out different biological roles from sense transcripts. Huge numbers of unannotated transcripts, which may include *cis*-NATs previously identified by gene structure predictions [19], are not supported by RAP. Expression of the predicted transcripts from mapped reads was validated by qRT-PCR in our previous study [21, 22]. Transcribed extensions and variants from close genes might also have been included. Considering the above, these *cis*-NATs transcripts are possibly novel transcripts responsive to Cd exposure in rice, and may include non-protein coding transcripts, novel protein transcripts, transcripts specifically responsive to Cd, or even transcripts that may have lethal functions in *E. coli*. Analysis of variance (G-test {FDR < 0.01}) on the RPKM-derived read counts was used to dissect the responses of *cis*-NATs based on single RAP gene loci associated with the time of collection (0, 1, 12 and 24 h), hydroponics type (control and Cd-treated) and tissue (root and shoot) (Fig. 3). In roots, the number of upregulated *cis*-NATs gradually increased and reached a maximum under 50 μM Cd at 24 h. This tendency was different from the downregulated transcripts in roots and shoots, and the upregulated transcripts in shoots. The number of upregulated *cis*-NATs originating from single loci annotated by RAP at 24 h was 14, 33, 38 and 409 in roots and 5, 8, 6 and 62 in shoots after 0, 0.2, 1 and 50 μM Cd exposure, respectively, compared with non-treatment (0 μM at 0 h) (Fig. 3). Because more *cis*-NATs responded to the stress at 24 h as the Cd concentration increased, the expression of the *cis*-NATs was affected by the degree of stress. The number of responsive RAP transcripts was much higher than that of *cis*-NATs (Fig. 2), suggesting that *cis*-NATs might be rare and respond to specific conditions in rice.

### Functional characterization of RAP transcripts with *cis*-NATs upregulated under Cd exposure

**Fig. 4:**
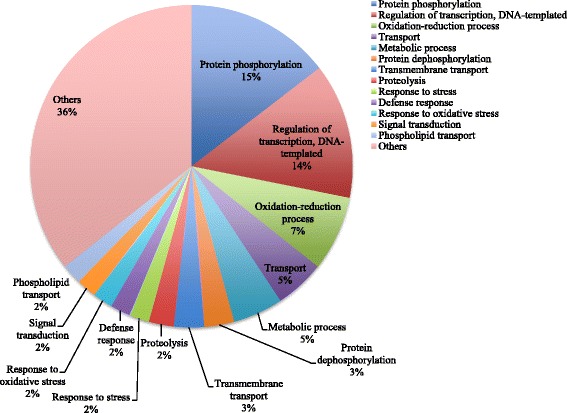
Distribution of Gene Ontology (GO) biological process categories for RAP transcripts with *cis*-NATs upregulated under Cd exposure. The percentages of transcripts upregulated in roots after 24 h of 50 μM Cd exposure in different GO categories are summarized

To determine whether the RAP transcripts with *cis*-NATs had functional tendencies, functional annotations were obtained from the Gene Ontology (GO) database. Functional annotations were assigned to the 409 RAP transcripts with upregulated *cis*-NATs in roots at 24 h under 50 μM Cd treatment (Fig. 4), because this was the maximum number among the time points (Fig. 3, Table 2). The major categories included protein phosphorylation (GO:0006468), regulation of transcription, DNA-templated (GO:0006355), oxidation-reduction process (GO:0055114) and metabolic process (GO:0008152), a pattern similar to the general stress response. Transcripts for transport (GO:0006810) and transmembrane transport (GO:0055085) were also detected, which clearly validated the RNA-Seq expression profiling data obtained from the rice tissues under Cd exposure. The upregulation of transport transcripts under Cd exposure corresponded with the results in Table 1. The functional categories of the GO classifications were similar between the upregulated RAP transcripts and RAP transcripts with upregulated *cis*-NATs in roots at 24 h under Cd exposure (Additional file 2: Figure S1). This suggests that the RAP transcripts with upregulated *cis*-NATs might not have any specific functional tendency compared with the functions of the upregulated RAP transcripts under Cd exposure.

**Table 2:** Cadmium-upregulated *cis*-NATs identified in roots by ssRNA-Seq analysis

		Root														
		Antisense transcript							RAP							
		Fold Change			RPKM				Fold Change			RPKM				
		Cd_50 μM			Control		Cd_50 μM		Cd_50 μM			Control		Cd_50 μM		
Transcript	Length (bp)	1 h	12 h	24 h	0 h	1 h	12 h	24 h	1 h	12 h	24 h	0 h	1 h	12 h	24 h	RAP Description
*Os02t0108500-01*	985	38.5	233.6	204.4	1.6	99.9	610.9	534.6	1	1.6	2.6	0.0	0.0	0.6	1.6	Non-protein coding transcript
*Os02t0181200-00*	393	156	136.9	102.7	4.6	868.2	761.4	571.0	1	1	1	0.0	0.0	0.0	0.0	Hypothetical protein
*Os01t0949800-01*	857	9.1	49.5	60.3	0.0	8.1	48.5	59.3	0.1	0.1	0.1	455.2	42.7	32.2	35.9	OsGSTU36
*Os11t0474100-01*	776	1.8	19.7	42.7	0	0.8	18.71	41.7	0.3	0.7	0.9	9.26	2.56	6.34	8.54	Conserved hypothetical protein
*Os05t0568000-01*	2355	16.9	89.2	40.4	0.0	15.9	88.2	39.4	3.6	1.3	0.8	91.3	331.7	116.3	73.0	Fatty acid elongase (FAE)1
*Os01t0795600-01*	555	1.2	20.4	38.5	0.0	0.2	19.4	37.5	10.6	43.8	48.6	2.5	36.2	153.4	170.2	Conserved hypothetical protein
*Os04t0407500-00*	913	2.4	57.4	34.5	0.0	1.4	56.4	33.5	0.5	0.7	0.5	3.3	1.1	1.8	1.3	Unknown protein
*Os09t0500151-00*	492	3.5	31.7	33.8	0	2.52	30.7	32.75	0.7	0.7	0.7	0.41	0	0	0	Hypothetical genes
*Os01t0661750-00*	354	1.3	28.6	31.5	0.6	1.1	43.7	48.1	1	1	1	0.0	0.0	0.0	0.0	Hypothetical conserved gene
*Os05t0182000-01*	843	1.2	9.6	23.7	0.0	0.2	8.6	22.7	0.6	0.6	0.4	5.2	2.5	2.6	1.7	Conserved hypothetical protein
*Os12t0153700-00*	1119	0.8	6.2	22.1	0.71	0.44	9.52	36.81	1	1	1	0	0	0	0	Peptidase aspartic, catalytic domain containing protein
*Os11t0536400-00*	165	10	17.3	19.1	0	9.03	16.32	18.11	1	1	1	0	0	0	0	Non-protein coding transcript
*Os07t0545800-01*	2161	1.1	18.3	18.6	0	0.11	17.28	17.56	6.8	5.2	3.6	26.5	186.02	141.02	97.53	GRAS domain family protein
*Os11t0429433-00*	687	1	10.2	17.5	0	0	9.2	16.46	0.6	0.7	0.9	7.84	4.34	4.77	7	Hypothetical gene
*Os08t0183700-00*	276	1.5	6.1	17.5	0	0.45	5.09	16.48	1	1	1	0	0	0	0	–
*Os10t0545800-01*	1162	1.1	9.9	17.4	0	0.11	8.87	16.44	9.7	20.8	16.2	7.9	85.77	184.37	143.03	Nucleic acid-binding, OB-fold domain containing protein
*Os01t0783600-01*	866	8.5	24.7	17.4	0.5	11.5	35.0	24.5	0.5	0.3	0.2	18.7	8.0	5.4	3.0	Frataxin
*Os08t0153750-00*	472	1.7	11.5	16.8	4.23	7.63	59.28	86.72	1	1	1	0	0	0	0	Hypothetical protein
*Os05t0142600-00*	246	1	2.4	14.7	0.0	0.0	1.4	13.7	3.5	29.2	32.2	4.1	16.7	146.6	162.2	Hypothetical protein
*Os02t0142060-01*	877	1.3	8.5	14.6	0.0	0.3	7.5	13.6	0.3	0.4	0.3	3.2	0.4	0.7	0.2	Conserved hypothetical protein
*Os12t0219300-00*	303	2.3	8.2	13.9	1.98	5.73	23.57	40.31	1	1	1.9	0	0	0	0.86	–
*Os03t0227500-01*	474	1	8.4	13.6	0.0	0.0	7.4	12.6	0.9	0.7	0.9	3.4	2.9	2.0	2.7	Hypothetical protein
*Os03t0231400-01*	781	2.4	13	12.9	2.6	7.6	45.1	44.8	0.4	1.1	1.1	3.6	0.8	3.9	4.0	Hypothetical protein
*Os05t0133900-02*	2694	2.2	5.2	12.3	0.1	1.3	4.6	12.1	0.7	0.5	0.6	13.2	8.9	5.8	7.4	DNA methyl transferase 106
*Os01t0159800-01*	1473	0.9	5.4	12.3	0.3	0.1	5.9	14.6	0.7	2.3	3.1	8.1	5.1	19.6	27.0	DNA binding protein (bHLH)
*Os01t0223600-01*	1730	1	12	11.4	0.2	0.3	13.7	13.0	0.6	0.3	0.3	42.9	25.3	13.8	11.3	Pto kinase interactor 1-like protein
*Os05t0194500-01*	1510	1.3	10.5	11.1	0.0	0.3	9.5	10.1	2.2	9.2	9.3	3.2	8.2	37.3	37.6	DNA binding protein (NAM)
*Os03t0693400-01*	787	1.6	6.1	10.9	0.0	0.6	5.1	9.9	0.7	0.5	0.8	33.7	23.5	17.9	27.6	Unknown protein
*Os01t0955400-00*	453	1	4.4	10.5	0.0	0.0	3.4	9.5	15.8	32.5	35.1	0.0	14.8	31.5	34.1	OsCML23
*Os09t0392700-01*	844	1	2.8	10.4	0	0	1.8	9.39	0.6	1	1.9	2.84	1.32	2.77	6.16	Hypothetical conserved gene
*Os03t0180100-01*	885	1.3	9.1	10.3	0.0	0.3	8.1	9.3	1.8	1.3	1	12.9	23.7	17.3	13.1	Unknown protein
*Os05t0553900-00*	312	1	3.3	10.2	0.0	0.0	2.3	9.2	1	1	1.8	0.0	0.0	0.0	0.8	–
*Os12t0433066-00*	822	1.6	9.6	9.8	1.46	2.87	22.5	23.08	0.8	0.9	1	0.49	0.15	0.28	0.47	Hypothetical gene
*Os06t0147100-01*	698	2.4	11.4	9.5	0.57	2.84	16.94	13.96	0.4	1.4	2.1	104.06	37.52	143.4	220.61	Conserved hypothetical protein
*Os11t0121000-00*	947	1.4	10.9	9.4	0	0.39	9.89	8.37	1.5	1.4	1.6	2.95	4.98	4.57	5.35	Hypothetical conserved gene
*Os06t0181800-00*	1174	1	8.7	8.9	0	0	7.68	7.86	3.9	6.7	6.9	2.04	10.78	19.25	20.03	Unknown protein
*Os05t0121600-00*	985	1.1	5	8.9	0.0	0.1	4.0	7.9	1.3	0.9	0.8	111.0	140.9	99.8	88.5	AP2/EREBP
*Os08t0104100-01*	998	1.5	6.6	8.8	0	0.5	5.63	7.81	0.6	0.3	0.2	53.78	33.45	14.31	12.63	Conserved hypothetical protein
*Os05t0209500-01*	685	1	6	8.8	0.0	0.0	5.0	7.8	1.3	1.7	2	16.3	21.9	27.7	33.2	Conserved hypothetical protein
*Os02t0819500-04*	1080	1	13.8	8.8	0.0	0.0	12.8	7.8	0.6	0.6	0.8	56.7	31.5	33.7	45.0	Cysteine-type peptidase
*Os08t0474600-01*	1707	1	9.2	8.7	0	0	8.16	7.69	0.3	0.6	0.5	9.59	2.18	5.01	4.26	Heat shock protein DnaJ
*Os07t0561650-00*	504	1	6.1	8.5	0	0	5.11	7.48	1	1	1	0	0	0	0	–
*Os09t0442600-01*	2186	1.3	8	8.4	0	0.28	7.02	7.37	1.2	1.1	1.2	47.37	55.47	52.43	57.36	Plastid (P)ppGpp synthase
*Os11t0307600-01*	1013	1.3	7.5	8.3	0	0.25	6.47	7.31	0.3	0.2	0.3	12.61	3.31	2.2	2.69	–
*Os01t0771200-01*	963	0.5	4.7	8.3	1.5	0.1	10.6	19.4	1.3	2.3	2.7	28.6	37.5	65.7	80.0	Mal d 1-associated protein
*Os06t0133400-01*	490	1	5.3	8.2	0	0	4.3	7.16	16.8	18.2	23.6	17.92	317.62	343.09	445.26	Conserved hypothetical protein
*Os01t0118700-01*	2087	0.8	5.5	8.2	0.2	0.0	5.5	8.8	2.9	2.6	2.8	24.0	71.3	63.3	68.9	HGA4
*Os06t0580100-02*	2283	1.1	6	8.1	0	0.05	5.03	7.11	0.3	0.4	0.5	46.67	12.45	18.15	23.85	Hypothetical gene
*Os01t0908200-01*	1446	1.1	5.5	8.1	0.0	0.1	4.5	7.1	1	0.3	0.4	60.0	58.9	19.5	21.4	Zinc finger (TAZ-type)
*Os09t0251900-00*	384	1	7.7	7.8	0	0	6.71	6.77	1	1	1	0	0	0	0	–

Reads were mapped to the rice genome and responsive cis-NATs were identified by G-tests. Top 50 of the upregulated cis-NATs in roots under 50 uM Cd exposure at 24 h are shown

### Characterization of the expression of RAP transcripts with *cis*-NATs under Cd stress

**Fig. 5:**
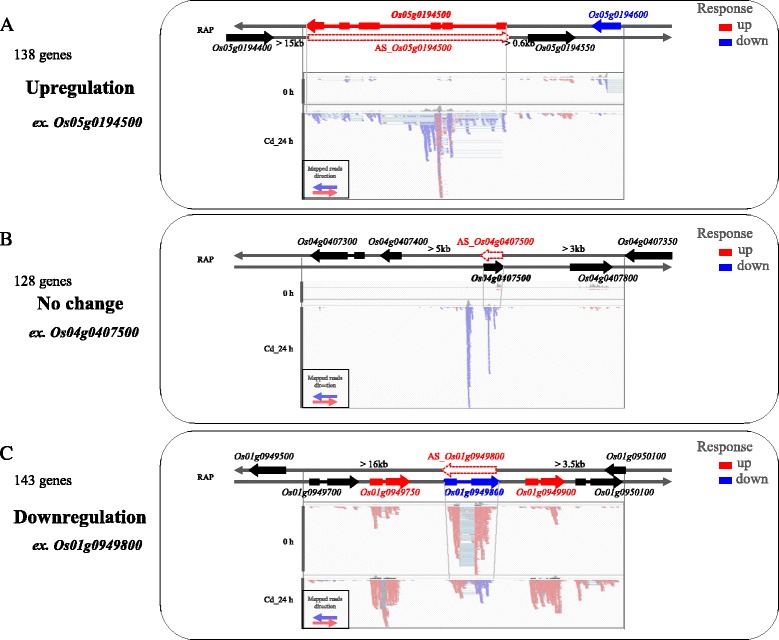
Examples of RAP transcripts with *cis*-NATs upregulated after Cd exposure for 24 h. The 409 RAP transcripts with antisense transcripts upregulated under Cd exposure were classified into three groups according to their expression changes: **a** upregulation (red arrow, ex. *Os05g0194500*), **b** no change (black arrow, ex. *Os04g0407500*) and **c** downregulation (blue arrow, ex. *Os01g0949800*). Dashed arrows show the schematic regions of the antisense transcripts AS_*Os05g0194500*, AS_*Os04g0407500* and AS_*Os01g0949800*, respectively. Reads (with their orientations; light blue and light red) were mapped to the rice genome as shown in the lower panel

The functions of RAP transcripts with upregulated *cis*-NATs did not show any specific tendency compared with RAP transcripts. Thus, we investigated the regulation pattern based on the responses of the 409 RAP transcripts with upregulated *cis*-NATs in roots at 24 h under 50 μM Cd exposure. The transcripts were classified into three sub-groups based on their expression under Cd exposure: 138 RAP transcripts with upregulated *cis*-NATs were upregulated (ex. *Os05t0194500*), 128 RAP transcripts with upregulated *cis*-NATs showed no change (ex. *Os04t0407500*) and 143 RAP transcripts with upregulated *cis*-NATs were downregulated (ex. *Os01t0949800*) (Fig. 5, Additional file 1: Table S2). The upregulation of *Os05t0194500* (*ONAC085*) means that few reads in the sense direction (light blue arrows) were mapped to the *Os05g0194500* locus before treatment (0 h) but the number of reads increased drastically under Cd exposure. While few reads in the antisense direction (light red arrows) were mapped to the *Os05g0194500* locus (*AS_Os05t0194500-0* as antisense transcripts with sequence complementarity to *Os05t0194500-01* on the opposite DNA strand) at 0 h, they also increased at 24 h under Cd exposure (Fig. 5a). This group included many genes encoding regulatory proteins such as Os01g0749300 (HSFA4A) that confer Cd tolerance [23], and they may function in regulating downstream genes under Cd exposure. Several RAP genes with upregulated *cis*-NATs encoding functional proteins such as transporters and protective macromolecules (enzymes, protein complexes and membranes) were also included in the group. For example, the gene encoding metal ion transporter (*Os04t0533900*) may function in efflux pumping of Cd at the plasma membrane and uptake of Cd from the soil through the root. The gene encoding sugar transporter (*Os03t0218400*), a homolog of *Arabidopsis STP13* (*At5g26340*) [24], may function in transporting sugars through plasma membranes to help adjust carbon and nitrogen metabolism during plant growth and development. The gene encoding lipase (*Os12t0554500*) may function to change the lipid composition of membranes or function to repair stress-induced damage to membranes by regulating permeability to toxic ions and the fluidity of the membrane [25, 26]. The upregulation of their *cis*-NATs may contribute to enhancing the expression of the opposite RAP transcripts. The invariability of *Os04t0407500* (*unknown protein*) means that a few reads in the sense direction (light red arrows) were mapped to the *Os04g0407500* locus both at 0 h and under Cd exposure. While few reads in the antisense direction (light blue arrow) were mapped to the *Os04g0407500* locus (*AS_Os04t0407500* as antisense transcripts with sequence complementary to *Os04t0407500* on the opposite DNA strand) at 0 h, they drastically increased at 24 h under Cd exposure (Fig. 5b). The downregulation of *Os01t0949800* (*Glutathione S-transferase GST 28*: *GSTU36*) means that many reads in the sense direction (light red arrows) were mapped to the *Os01g0949800* locus before treatment (0 h) but the reads decreased drastically under Cd exposure. While few reads with antisense direction (light red arrows) were mapped to the *Os01g0949800* locus (*AS_Os01t0949800* as antisense transcripts with sequence complementary to *Os01t0949800* on the opposite DNA strand) at 0 h, they increased at 24 h under Cd exposure (Fig. 5c). The GST enzyme catalyzes the conjugation of glutathione to a range of electrophilic substrates for detoxification and protection of the cell [27]. The downregulation of *GSTU36* in roots under Cd exposure was consistent with our previous report [19], suggesting it probably functions during normal growth to maintain homeostasis in rice. Functional genes related to plant growth including plant hormone metabolism include genes such as *IAA11* (*Os05t0559400*), which functions in lateral root initiation [28], and *ARF23* (*Os11t0523800*), which functions in auxin-mediated cell growth by promoting RMD (rice actin-binding protein) expression [29], were also included in this group. The transcriptional genes in this category were negatively correlated with *cis*-NAT accumulation. *Cis-*antisense overlapping pairs have the potential to generate nat-small RNAs, which originate from double-stranded RNA molecules [30], whose action might be contributing to the correlation with quick transcriptional changes, especially in response to stress.

The loci of the *cis*-NATs with sequence complementary to RAP genes on the opposite DNA strand were more than 3 kb away from the adjacent RAP genes (Fig. 5). Although the expression of *cis*-NATs complementary to RAP genes on the opposite DNA strand changed under Cd exposure, it is difficult to define the exact lengths of the *cis*-NATs and their loci because they may include splicing isoforms and variants of adjacent genes. Our data clearly indicated that the 409 RAP transcripts with upregulated *cis*-NATs in roots after 24 h of 50 μM Cd exposure could be roughly divided into three sub-groups, suggesting that the expression of each RAP transcript might not always be correlated with the upregulated *cis*-NAT. ssRNA-Seq analysis revealed novel transcripts that were responsive to some degree (ex. ≈ 1%: 409 RAP transcripts with upregulated *cis*-NATs/38,123 expressed RAP genes in roots at 24 h under 50 μM Cd exposure), suggesting that this method can contribute to gene annotation and improve our understanding of the transcriptome network in rice.

### Identification of stress-specific *cis*-NATs under cold and ABA treatments

**Fig. 6:**
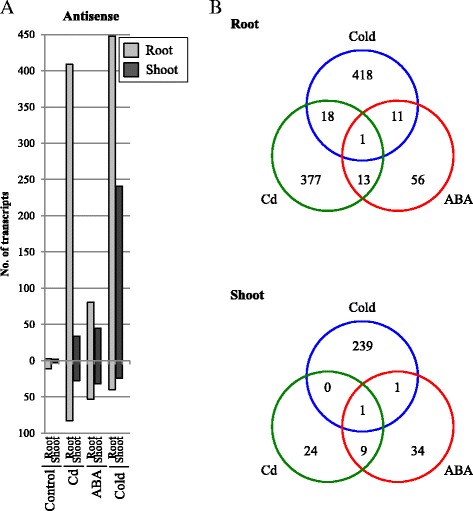
Distribution of upregulated and downregulated RAP transcripts with corresponding upregulated or downregulated *cis*-NATs under Cd, ABA, cold, and control treatments for 24 h. **a** RPKM fold changes at 24 h were calculated for treated samples compared with non-treated samples (0 h) in roots (light gray) and shoots (gray). The total numbers of upregulated (upper) and downregulated (lower) transcripts in roots and shoots identified by ssRNA-Seq were determined by a G-test (FDR < 0.01). **b** The Venn diagram shows the number of RAP transcripts with antisense transcripts upregulated under Cd exposure (green), ABA treatment (red), and cold treatment (blue)

Generally, many genes show specific expression under abiotic stresses, so the responsive *cis*-NATs were investigated under cold stress and ABA treatment, and in a control after 24 h. The *cis*-NATs showed greater up- or downregulation in roots compared with shoots in all conditions including the control (Fig. 6a). Compared with non-treatment, the *cis*-NATs were more responsive under stress conditions than in the control after 24 h. In particular, cold-stress conditions had the biggest effect on the *cis*-NAT expression profile, with the most upregulated transcripts in roots and shoots among the conditions (Fig. 6a). Interestingly, the number of RAP transcripts upregulated under cold stress was lower than that under Cd and ABA, suggesting that the expression tendencies of RAP transcripts and *cis*-NATs were diverse with regard to stresses (Fig. 6a, Additional file 2: Figure S2A). The number of up- or downregulated RAP transcripts in shoots was greater than that in roots in some conditions (Additional file 2: Figure S2A). Venn diagram analysis of the upregulated *cis*-NATs among stress conditions showed that most were expressed specifically in one condition in both roots and shoots (Fig. 6b). Additionally, 7.8% (32/409) of the upregulated *cis*-NATs under Cd in roots and 2.9% (10/34) of those in shoots were commonly upregulated by cold and/or ABA. Only one *cis*-NAT was upregulated under all stress conditions in both roots and shoots (Fig. 6b). Additionally, 54.7% (1546/2825) of the upregulated RAP transcripts under Cd in roots and 64.7% (2892/4471) of those in shoots were commonly upregulated by cold and/or ABA (Additional file 2: Figure S2B). Meanwhile, 53.0% (44/83) of the downregulated *cis*-NATs under Cd in roots and 57.1% (16/28) of those in shoots, and 68.5% (9418/13,742) of the downregulated RAP transcripts under Cd in roots and 89.3% (4326/4844) of those in shoots were commonly downregulated by cold and/or ABA (Additional file 2: Figure S3A, B). This indicated that, like the RAP transcripts, the *cis*-NATs identified by ssRNA-Seq analysis had stress-specific responses.

## Conclusion

Our data encompass the expression of *cis*-NATs with sequence complementarity to RAP genes on the opposite DNA strand. The *cis*-NAT loci were defined by their transcriptional structures as revealed by ssRNA-Seq, but were not supported by RAP. Many RAP transcripts possessed *cis*-NATs, which can be classified as novel transcripts in rice. This study also revealed *cis*-NATs that were responsive to Cd exposure to some extent and some that were stress-specific. The numbers of responsive *cis*-NATs and RAP transcripts on the opposite strand both changed depending on the combination of tissue, Cd concentration and time point. Future analysis of the exact loci of the transcribed *cis*-NATs and the timing of their expression will provide useful knowledge for understanding the transcriptome network in rice under Cd exposure.

## Additional files

Additional file 1: **Table S1.** Mapping of RNA-Seq reads obtained from root and shoot samples to the reference IRGSP-1.0 genome sequence. **Table S2.** Classification of 409 RAP transcripts with upregulated *cis*-NATs into three groups based on the expression of transcribed RAP transcripts from the opposite DNA strand. (XLS 367 kb)

Additional file 2: **Figure S1.** Distribution of Gene Ontology (GO) biological process categories for RAP transcripts upregulated under Cd exposure. The percentages of upregulated transcripts in roots after 24 h of 50 μM Cd exposure in different GO categories are summarized. **Figure S2.** Distribution of upregulated RAP transcripts. (A) The numbers of upregulated and downregulated RAP transcripts under Cd, ABA and cold treatments, and in the control after 24 h in roots (light gray) and shoots (dark gray). RPKM fold changes at 24 h were calculated for treated samples compared with non-treated samples (0 h). The total numbers of upregulated (upper) and downregulated (lower) transcripts in roots and shoots identified by ssRNA-Seq were determined by a G-test (FDR < 0.01). (B) Venn diagram showing the RAP transcripts upregulated under Cd exposure (green), ABA treatment (red), and cold treatment (blue). **Figure S3.** Venn diagram analysis of downregulated RAP transcripts. The numbers of RAP transcripts with *cis*-NATs (A) and RAP transcripts (B) downregulated under Cd exposure (green), ABA treatment (red), and cold treatment (blue) are shown. (PPTX 57 kb)

## Abbreviations

Cd: Cadmium
*cis*-NAT: *cis*-natural antisense transcript
FLC: FLOWERING LOCUS C, floral repressor gene
GO: Gene ontology
IRGSP-1.0: The International Rice Genome Sequencing Project-1.0
PHO1;2: PHOSPHATE1;2)
RAP: Rice annotation project
RPKM: Reads Per Kilobase exon Model per Million mapped reads
ssRNA-Seq: Strand-specific RNA-Seq
