# Supplementary material for: Genome-wide analysis of rice cis-natural antisense transcription under cadmium exposure using strand-specific RNA-Seq
Source: BMC Genomics. 2017 Oct 6;18:761. doi: 10.1186/s12864-017-4108-5 (PMC6389181; doi:10.1186/s12864-017-4108-5)
Supplement: Supplementary file 2 — Figure S1. Distribution of Gene Ontology (GO) biological process categories for RAP transcripts upregulated under Cd exposure. The percentages of upregulated transcripts in roots after 24 h of 50 μM Cd exposure in different GO categories are summarized. Figure S2. Distribution of upregulated RAP transcripts. (A) The numbers of upregulated and downregulated RAP transcripts under Cd, ABA and cold treatments, and in the control after 24 h in roots (light gray) and shoots (dark gray). RPKM fold changes at 24 h were calculated for treated samples compared with non-treated samples (0 h). The total numbers of upregulated (upper) and downregulated (lower) transcripts in roots and shoots identified by ssRNA-Seq were determined by a G-test (FDR < 0.01). (B) Venn diagram showing the RAP transcripts upregulated under Cd exposure (green), ABA treatment (red), and cold treatment (blue). Figure S3. Venn diagram analysis of downregulated RAP transcripts. The numbers of RAP transcripts with cis-NATs (A) and RAP transcripts (B) downregulated under Cd exposure (green), ABA treatment (red), and cold treatment (blue) are shown. (PPTX 57 kb) [file 12864_2017_4108_MOESM2_ESM.pptx]

## Slide 1
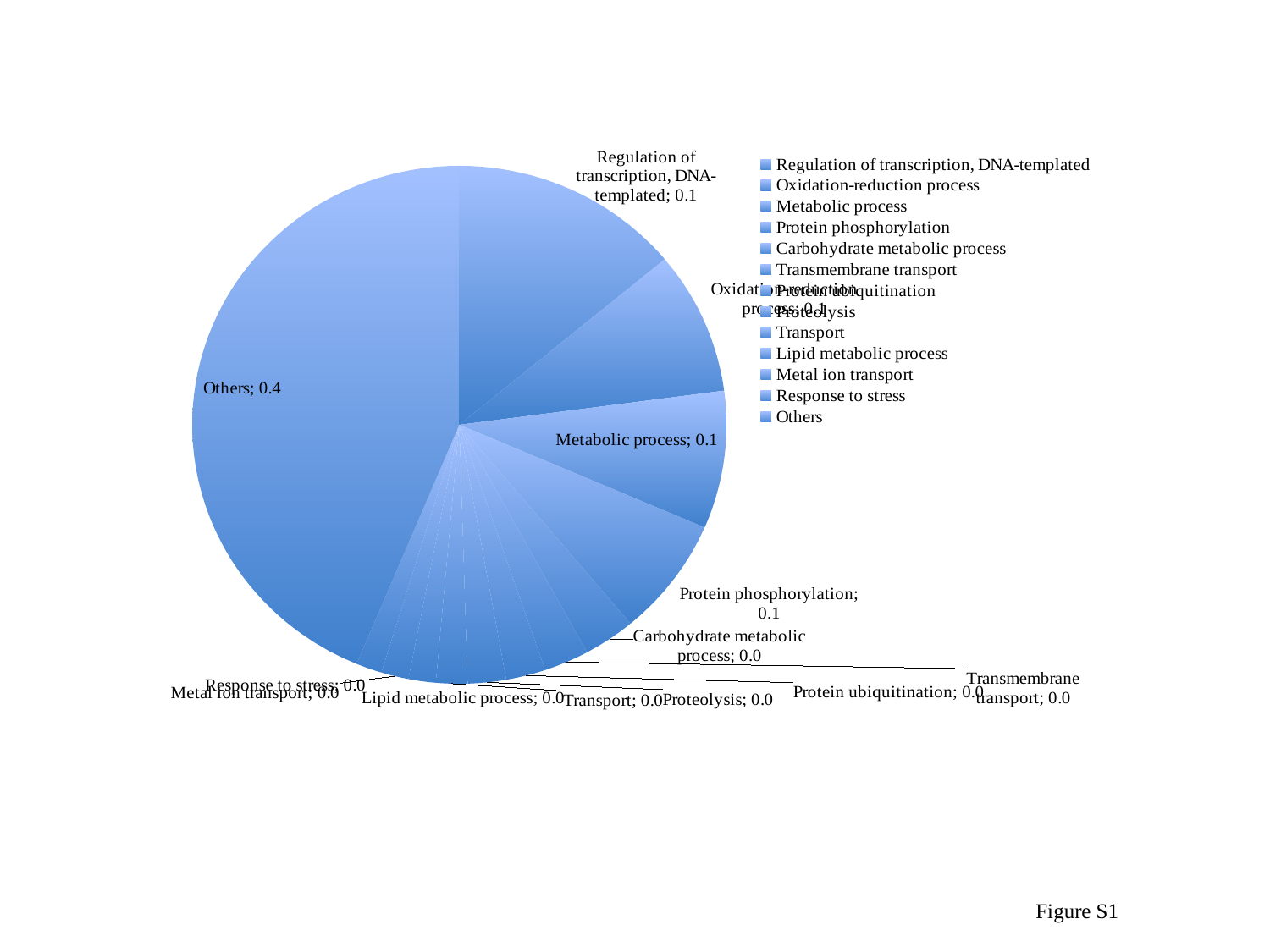

### Chart
| Category | % |
|---|---|
| Regulation of transcription, DNA-templated | 13.96694214876033 |
| Oxidation-reduction process | 8.92561983471075 |
| Metabolic process | 8.59504132231405 |
| Protein phosphorylation | 7.43801652892562 |
| Carbohydrate metabolic process | 3.140495867768595 |
| Transmembrane transport | 2.727272727272727 |
| Protein ubiquitination | 2.396694214876033 |
| Proteolysis | 2.314049586776858 |
| Transport | 1.900826446280992 |
| Lipid metabolic process | 1.652892561983471 |
| Metal ion transport | 1.652892561983471 |
| Response to stress | 1.570247933884298 |
| Others | 43.71900826446281 |Figure S1

## Slide 2
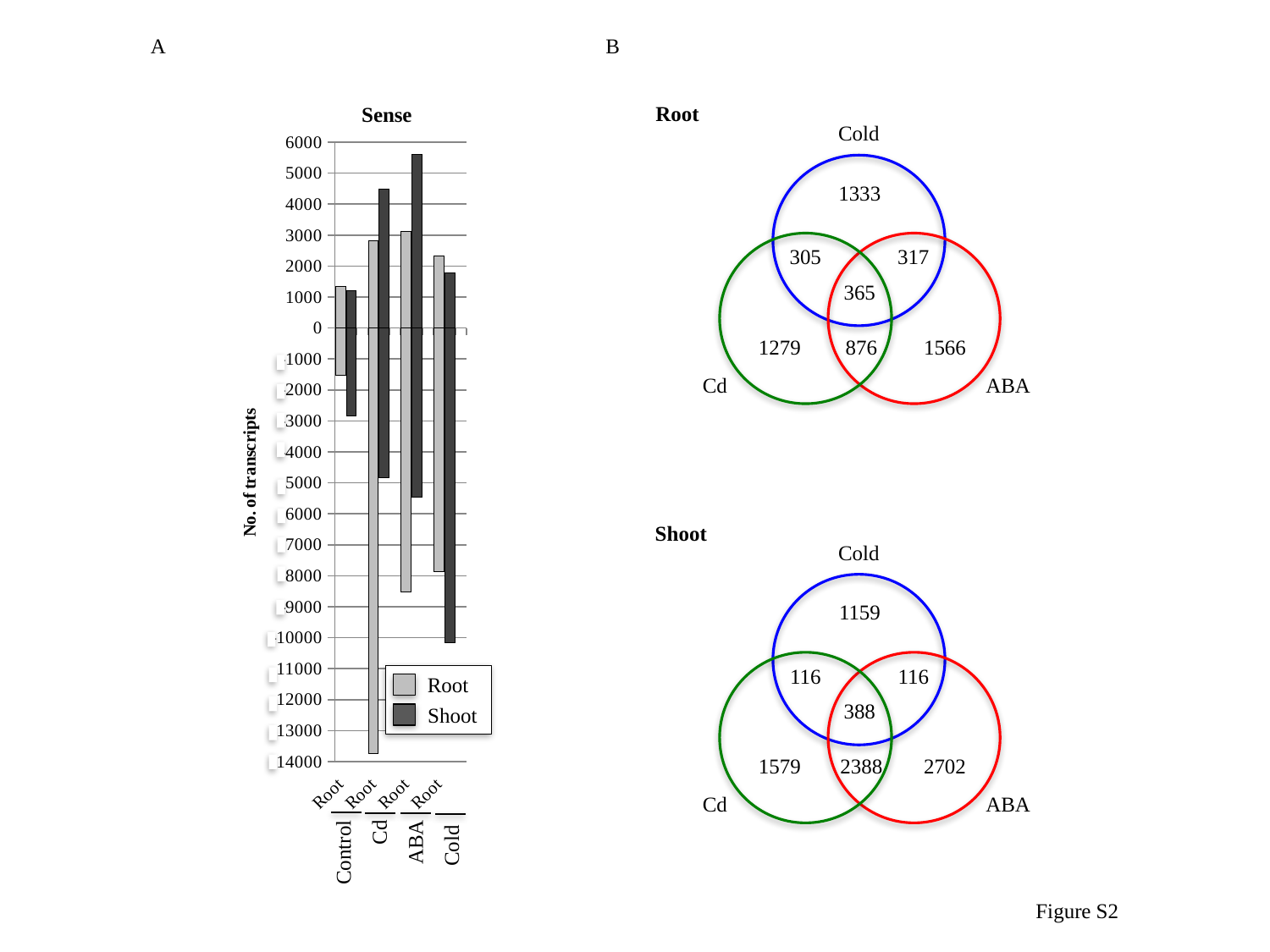

A
B
Root
### Chart: Sense
| Category | | |
|---|---|---|
| Root | 1333.0 | -1531.0 |
| Shoot | 1207.0 | -2836.0 |
| | None | None |
| Root | 2825.0 | -13742.0 |
| Shoot | 4471.0 | -4844.0 |
| | None | None |
| Root | 3124.0 | -8526.0 |
| Shoot | 5594.0 | -5471.0 |
| | None | None |
| Root | 2320.0 | -7874.0 |
| Shoot | 1779.0 | -10161.0 |
Root
Shoot
Cd
ABA
Cold
Control
Cold
1333
305
317
365
1279
876
1566
Cd
ABA
Shoot
Cold
1159
116
116
388
1579
2388
2702
Cd
ABA
Figure S2

## Slide 3
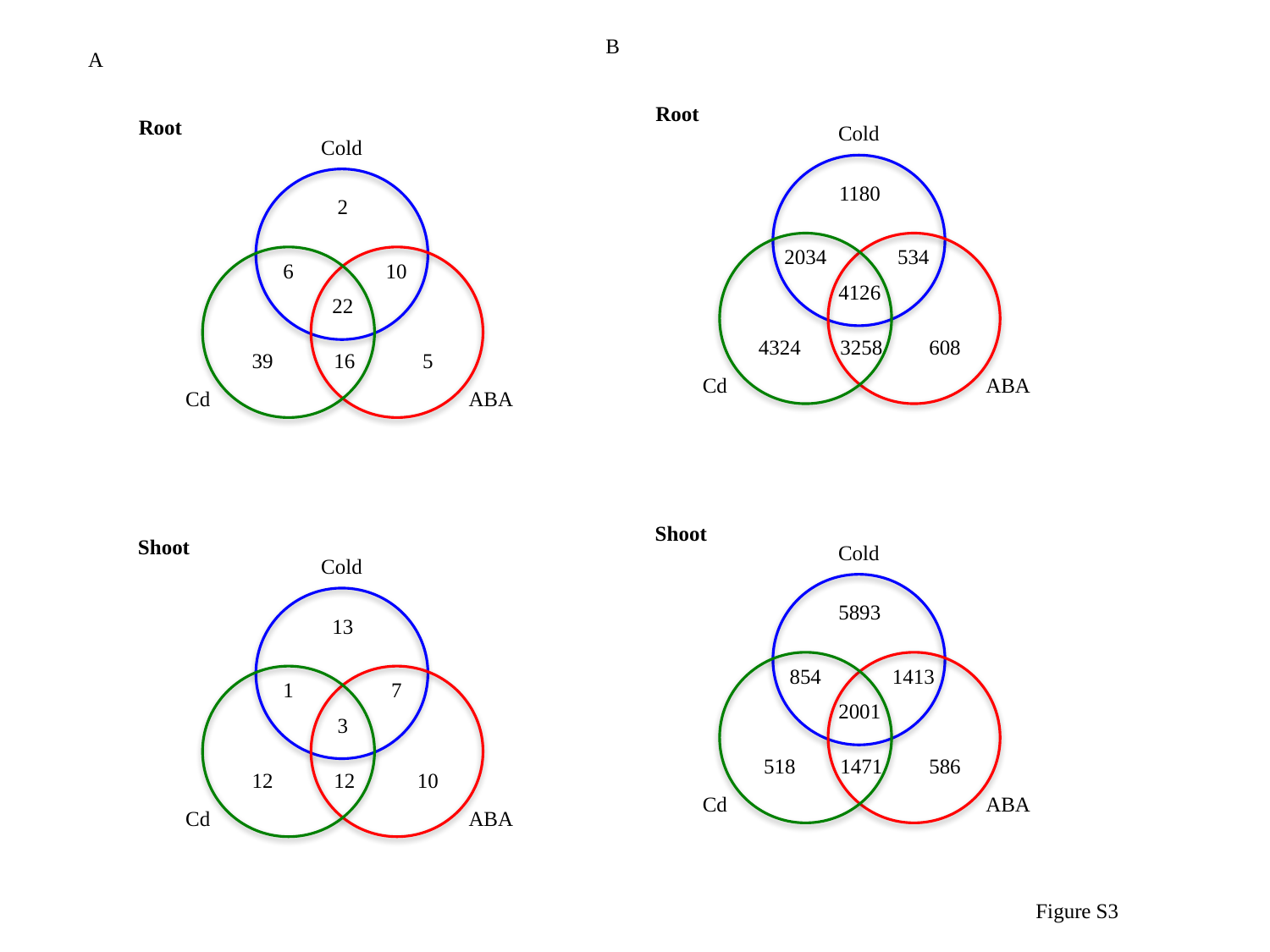

B
A
Root
Root
Cold
1180
2034
534
4126
4324
3258
608
Cd
ABA
Cold
2
6
10
22
39
16
5
Cd
ABA
Shoot
Shoot
Cold
5893
854
1413
2001
518
1471
586
Cd
ABA
Cold
13
1
7
3
12
12
10
Cd
ABA
Figure S3
